# Phenotypic characterization, molecular typing, and clonal relatedness within *Staphylococcus* isolates in healthy ocular conditions

**DOI:** 10.1007/s42770-026-01999-5

**Published:** 2026-06-24

**Authors:** Marco Finocchiaro, Nunziatina Russo, Nunzio A. Fazio, Joanna Gajewska, Giuseppe Scalia, Wioleta Chajęcka-Wierzchowska, Alessandra Pino, Cinzia Caggia, Cinzia L. Randazzo

**Affiliations:** 1 Department of Agriculture, Food, and Environment (Di3A), University of Catania, Via Santa Sofia 100, Catania, 95123 Italy; 2 Department of Industrial and Food Microbiology, University of Warmia and Mazury in Olsztyn, Plac Cieszyński 1, Olsztyn, 10-726 Poland; 3 ASP Catania, U.O.C. of Ophthalmology Acireale, Paternò, CT Italy; 4 NANOMED-Research Center in Pharmaceutical Nanomedicine and Nanotechnologies, University of Catania, Viale A. Doria 6, Catania, 95125 Italy

**Keywords:** *Staphylococcus* spp., Antimicrobials, Multidrug resistance, PFGE profile

## Abstract

This study aimed to explore the culturable bacterial community in healthy volunteers, with a focus on the staphylococcal population. First, 120 bacterial isolates were collected from conjunctival swabs of 49 healthy volunteers (aged 18 to 77 years). Isolates were characterized by phenotypic analyses, and 68 of them were confirmed as staphylococci by MALDI-TOF/MS and a multiplex PCR assay. The *Staphylococcus* strains (*n* = 68) were tested for resistance to the most relevant clinical antibiotics. Clonal relationships among isolates were evaluated by pulsed-field gel electrophoresis (PFGE), and the presence of *mec*A and *mec*C, as well as *pvl* genes, was investigated. *Staphylococcus aureus* (74%) was the major identified species, followed by *Staphylococcus epidermidis* (19%) and *Staphylococcus xylosus* (7%). A high incidence of resistance was detected against gentamicin (64%), erythromycin (62%), and penicillin (51%). Moreover, 33 isolates (48%) exhibited multidrug-resistant phenotypes, and the two *S. epidermidis* strains harbouring the *mec* genes exhibited the highest resistance. A marked heterogeneity among isolates, mostly for *S. aureus* and *S. epidermidis*, was revealed, clustering them into 20 and 4 genotypes, respectively. The prevalence of the same clones, isolated from different volunteers, across the three species, and the different phenotypic resistance patterns within the same PFGE type, was observed. These findings corroborate that staphylococci constitute a significant component of the ocular microbiota in healthy individuals; however, their exhibited antimicrobial resistance profiles are of concern.

**Supplementary Information:**

The online version contains supplementary material available at 10.1007/s42770-026-01999-5.

## Introduction

The ocular surface is the interface between the eye and the environment, hosting a collection of microorganisms that constitute the ocular microbiota [1–4]. Although the existence of a distinct and stable ocular surface microbiota remains under investigation, current evidence suggests that the ocular surface harbors a microbial population poorer than those described in other mucosal environments, such as the gastrointestinal tract, oral cavity, and vaginal mucosa [4, 5]. The ocular microbiota includes both probiotics and opportunistic bacteria [6], grouped into fewer than 90 genera [7]. This microbiota, together with the tear film—which contains lactoferrin, defensins, and lysozyme—provides a protective role by preventing the colonization of pathogenic microorganisms [8–12]. Current evidence indicates that the cultivable microbiota of the conjunctiva and eyelid margins are predominantly composed of staphylococci, which are generally present at low abundance, followed by *Propionibacterium* spp. and *Corynebacterium* spp. At the same time, additional genera (*Acinetobacter*,* Brevundimonas*,* Pseudomonas*,* Aquabacterium*, and *Sphingomonas*) or some fungi are isolated less frequently [1, 13–15]. Recently, next-generation sequencing [16] has revealed the presence of both non-cultivable and transient microbiota [14, 15, 17]. However, despite the availability of multiple selective culture media [18], *Staphylococcus aureus* remains one of the most frequently detected species, being recovered from 20 to 80% of conjunctival specimens and 30–100% of eyelid specimens [1, 14, 19, 20]. This distribution aligns with the microbial profile characteristic of the eye community state type (ECST) [7]. The presence of *S. aureus* is worrying, as it is recognized as a leading cause of ocular and periocular infections, exhibiting rapid progression and accounting for nearly 70% of reported cases [21, 22]. The ability of this species to colonize the ocular niche, evading host defences, remains only partially understood, and efforts are being made to characterize ocular isolates and investigate virulence determinants [23].

Furthermore, a growing number of methicillin-resistant *S. aureus* (MRSA) isolates, as well as strains exhibiting reduced susceptibility to glycopeptides, have been associated with a wide range of ocular infections, including conjunctivitis, dacryocystitis, orbital cellulitis, keratitis, endophthalmitis, and postoperative infections [24, 25]. Among coagulase-negative staphylococci (CoNS), *Staphylococcus epidermidis* and *Staphylococcus hemolyticus*, along with *Staphylococcus saprophyticus* and *Staphylococcus lugdunensis* [26], are established commensals of the ocular surface; nevertheless, they have been implicated in ocular infections due to their capacity for biofilm formation and frequently observed antibiotic resistance [27–33], and *Staphylococcus xylosus*, generally considered non-pathogenic, has recently been associated with lacrimal duct infections in children [34].

The present study aimed to assess the biodiversity of culturable staphylococcal bacteria isolated from healthy volunteers of different ages. Pulsed Field Gel Electrophoresis (PFGE), *spa* typing, and antimicrobial resistance profiles were performed to determine the relatedness and the virulence of the most isolated strains.

## Materials and methods

### Subject recruitment and ethical clearance

A monocentric observational trial was performed at the Ophthalmology Complex Operative Unit of ASP Catania, Acireale Hospital (Italy). The study was conducted according to the Good Clinical Practices and the World Medical Association (WMA) policy regarding the Ethical Principles for Medical Research Involving Human Subjects, as stated in the Declaration of Helsinki. The protocol was approved by the Local Ethical Committee of Catania 2 (CEL, Catania 2), with the registration number 45/CEL.

Healthy volunteers were recruited, and written informed consent and past ocular history were obtained. The anterior segment ocular examination, along with a general medical examination, was performed on each patient undergoing conjunctival sampling. Patients with active inflammation or red eye were excluded.

### Applied selection criteria

To select a homogeneous group of subjects and assess individual variability within the conjunctival bacterial community, participants completed a questionnaire while specific inclusion and exclusion criteria were applied. The questionnaire included information regarding gender, age, and general and ocular health status. To qualify for participation, volunteers needed to meet certain criteria (inclusion criteria). They should not have a history of recent contact lens use, dry eye conditions, systemic diseases, ocular trauma or transplants, glaucoma, hypertension, uveitis, or retinal diseases. The selected volunteers had no medical history of systemic or ocular diseases, nor did they have any recent ocular trauma or transplants, glaucoma, hypertension, uveitis, or retinal diseases, or a recent (6-month) history of antibiotic treatment, or ocular or systemic medication use over the past year, including the use of corticosteroids and immunomodulatory drugs. Exclusion criteria were having had a disease to ocular surface disease/irritation, facial skin disease, use of probiotics or oral or topical antibiotics or prescription eye medication in the past 3 months, ocular surgery in the last 6 months, anaemias, active ocular infection, diabetes, pregnant or lactating women, vegetarians, and vegans, or an immunocompromised state.

### Sample collection and microbiological analysis

The healthy volunteers (*n* = 49) recruited in this study included males (*n* = 20) and females (*n* = 29), aged between 18 and 77 years. Samples from the tarsal and fornix conjunctivae from lower and upper lids were collected by swabbing two or three times by gently pressing on both eyes of each volunteer (Table 1).

**Table 1:** Generalities of subjects involved in the present study

Age	Female	Male
18–36	5	8
37–54	9	2
55–72	10	8
73–90	5	2
Total 49	**29**	**20**

Care was taken to avoid contact with the edge of the eyelid. Samples were collected using sterile cotton swabs, sealed in culture tubes containing 0.5 mL of modified Stuart’s transport media, and immediately transported to the microbiology laboratory at Di3A for the microbiological analyses. The conjunctival swabs were diluted in sterile saline solution and directly inoculated onto 5% Sheep Blood agar, Chocolate agar, Sabouraud Dextrose agar, Sulphite Polymyxin Sulphadiazine agar, Violet Red Bile Glucose agar, de Man and da Sharpe agar, Mannitol Salt agar, and Drigalski agar to count the culturable microorganisms, using standard microbiological techniques. The plates were incubated according to the manufacturer’s recommended conditions for each culture medium. Microbial growth was assessed daily, and colony-forming units were estimated after 7 days of incubation. All culture media were supplied by Liofilchem S.r.l. (Roseto degli Abruzzi, Italy).

### Bacterial isolation

Overall, microbial growth in the collected samples was scarce, with colonies predominantly recovered on Mannitol Salt agar. For each sample (one per subject), plates were examined across multiple culture media and all distinct colonies—prioritizing distinct morphotypes—were selected for further analysis. Recovered colonies were purified, microscopically examined, tested for catalase and oxidase activities, subjected to Gram staining, and subjected to biochemical characterization, including the ability to ferment various carbohydrates, before being stored at − 80 °C in liquid culture using 20% (v/v) glycerol.

### MALDI-TOF analysis

Following preliminary phenotypic characterization, isolates from each subject were grouped solely based on their phenotypic profiles. Bacteria with a distinctive pattern were subjected to identification by MALDI-TOF (Matrix-Assisted Laser Desorption/Ionization Time-of-Flight Mass Spectrometry) analysis with a VITEK MS instrument (bioMérieux, Marcy l’Etoile, France), as previously reported by Chajęcka-Wierzchowska and colleagues [35].

### Multiplex PCR

All staphylococci were subjected to PCR analysis to confirm their affiliation with *S. aureus* by amplifying the *spa* gene and virulence factors [36]. In detail, a multiplex PCR analysis was performed, as reported by Stegger and colleagues [37], to detect the *mec*A, *mec*C, and *pvl* genes. The primer sequences were reported in Table 1S. The *S. aureus* DMZ 683 and *S. aureus* DMZ 2569 were used as controls.

### Pulsed-field gel electrophoresis (PFGE) analysis

The clonal relationship among the 68 isolates was revealed by comparison of *Sma*I-digested profiles, using PFGE analysis [38]. The macro-restriction fragment separation was performed using the PulseNet standardized PFGE protocol for Molecular Typing of *S. aureus*. Digestion was carried out using the *Sma*I restriction enzyme for 4 h at 25 °C. The digested plugs were subjected to an electrophoretic run, using the CHEF-DR III system (Bio-Rad Laboratories, Hercules, CA, USA), in a 1.0% agarose gel in 0.5X TBE electrophoresis buffer at 6 V/cm, at 14 °C, with an initial switch time of 5 s, and a final switch time of 40 s for 21 h. Lambda ladder (New England BioLabs, Beverly, MA, UK) was run as a molecular weight marker (size range of 48.5–1018 Kb). *S. aureus* DMZ 683 and *S. aureus* DMZ 2569 were used as controls. After staining with gel red (Biotium, California), DNA bands were visualized by UV-transilluminator. PFGE images of gels were captured using the Gel Documentation System (Axygen Scientific, Torino, Italy). PFGE band patterns were generated by BioNumerics v. 7.6 software (Applied Maths, Sint-Martens-Latem, Belgium) with a tolerance position of 1%. Clustering was based on the unweighted pair group method with arithmetic averages (UPGMA). The Dice correlation coefficient was used to analyse the similarities of banding patterns.

### Antibiotic susceptibility test

The antimicrobial susceptibility test was performed using the Kirby-Bauer method. An inoculum of each strain, equivalent to 0.5 McFarland scale, was swabbed onto a Mueller-Hinton agar plate (Liofilchem srl). The antibiotic disc (Oxoid, Milan, Italy) was placed on the plate, and these incubated for 24 h at 37 °C. The inhibition zone and multidrug resistance (MDR), defined as acquired non-susceptibility to at least one agent in three or more antimicrobial categories relevant to the isolated species, were interpreted following the Clinical Laboratory Standards Institute (CLSI) guidelines [39]. The follow antibiotics were used: trimethoprim-sulfamethoxazole (SXT, 25 µg), quinupristine-dalfopristine (QD, 15 µg), penicillin (P, 10 µg), rifampicin (RD, 5 µg) linezolid (LZD, 30 µg), clindamycin (DA, 2 µg), levofloxacin (LEV, 5 µg), ciprofloxacin (CIP, 5 µg), gentamicin (CN 10 µg), and erythromycin (E, 15 µg). All antibiotics were purchased from Oxoid (Italy). Strains were categorized as susceptible (S), intermediate (I), or resistant (R), and *S. aureus* DMZ 2569 was used as the reference strain for antibiotic disc control.

## Results

### Screening of bacterial isolates

In the present study, 120 isolates were obtained from 49 volunteers in the different microbiological media. Based on a single phenotypic pattern, such as catalase-positive, oxidase-negative, positive Gram stain, and biochemical tests (oxidation/fermentation of carbohydrates), 100 isolates were presumptively ascribed to the *Staphylococcus* genus. The species affiliation was confirmed for 68 out of the 100 isolates by MALDI-TOF analysis, allowing discrimination among *S. xylosus*, *S. epidermidis*, and *S. aureus*. In detail, 5 *S. xylosus* strains were isolated from 3 subjects, 2 aged 49 and 1 aged 60. Moreover, 13 *S. epidermidis* strains were isolated from 11 subjects, mostly female, aged between 41 and 63. The remaining 50 isolates, identified as *S. aureus*, were obtained from 49 subjects of all ages.

In the multiplex PCR assay, it is noteworthy that the *mec*C determinant was detected in five *S. epidermidis* isolates, and concurrent carriage of *mec*A and *mec*C was observed in a single *S. epidermidis* isolate. By contrast, none of the *S. xylosus* isolates harbored *mec*A or *mec*C, and the *pvl* (Panton–Valentine leukocidin) gene was never detected (Table 2).

**Table 2:** PCR multiplex profile related to the detection of *spa*,* mec*A, *mec*C, and *pvl* genes among the 68 isolates belonging to *S. aureus* (*n* = 50), *S. epidermidis* (*n* = 13), and *S. xylosus* (*n* = 5)

Species	# of strains	Gene detection			
		spa	mecA	mecC	pvl
*S. aureus*	49	**+**	**-**	**-**	**-**
	1	**+**	**+**	**-**	**-**
*S. epidermidis*	8	**-**	**-**	**-**	**-**
	1	**-**	**+**	**+**	**-**
	4	**-**	**-**	**+**	**-**
*S. xylosus*	5	**-**	**-**	**-**	**-**

**Fig. 1:**
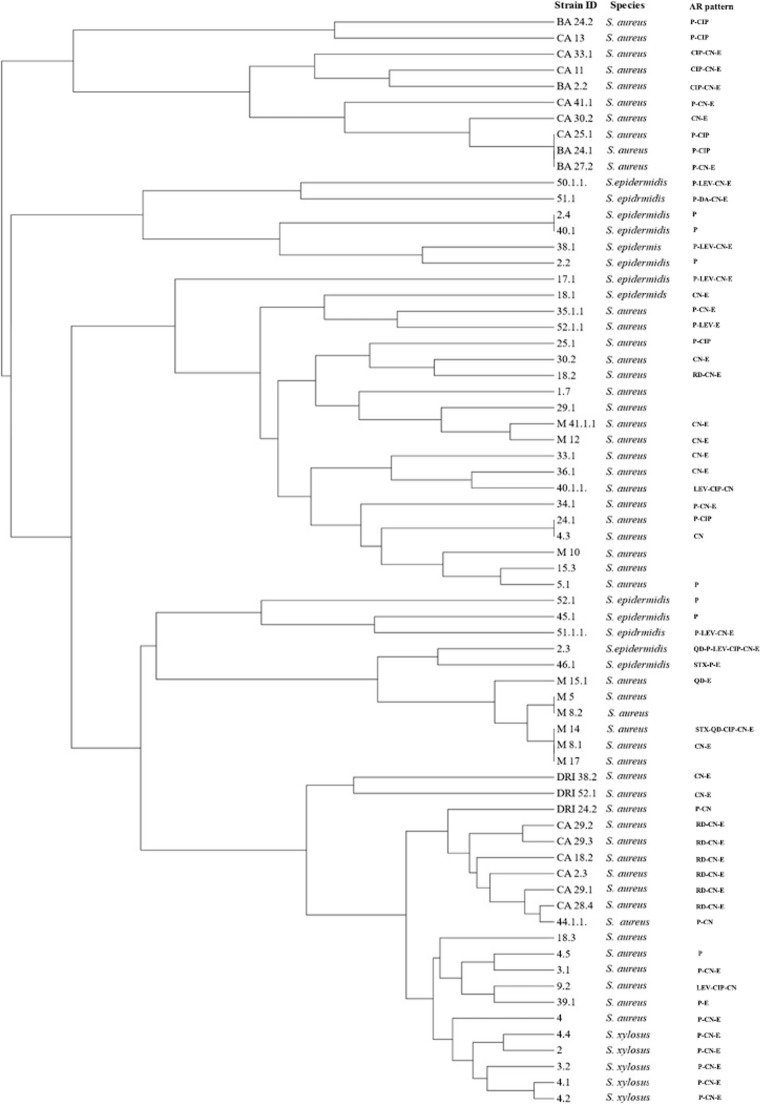
Staphylococci phylogenetic tree based on pulsed-field gel electrophoresis (PFGE) analysis and Antimicrobial Resistance pattern. Where not expressly indicated, the strains do not present antimicrobial resistance

Isolates of the three predominant staphylococcal species were genotyped by pulsed‑field gel electrophoresis (PFGE); and resulting patterns exhibiting greater than 81% similarity were interpreted as clonally related and consequently assigned to the same clone. The results allowed us to allocate the strains into different genotypes. In detail, the *S. epidermidis* isolates resolved into seven discrete clusters, each comprising one or two strains. By contrast, *S. aureus* exhibited substantially greater intraspecific diversity, resolving into 22 distinct clusters. The *S. xylosus* isolates were homogeneous in this analysis, coalescing into a single cluster (Fig. 1).

With respect to antimicrobial susceptibility across the ten antibiotics tested, all isolates except a single *S. aureus* strain exhibited resistance to at least one agent (Table 3).

**Table 3:** Antimicrobial susceptibility patterns of staphylococci (*n* = 68) isolated from the healthy ocular surface of patients

Antimicrobial class	Antimicrobial agent	*S. aureus* (*n* = 50)		*S. epidermidis* (*n* = 13)		*S. xylosus* (*n* = 5)		Total (*n* = 68)	
		*R* (%)	I (%)	*R* (%)	I (%)	*R* (%)	I (%)	*R* (%)	I (%)
Sulfonamides	Trimethoprim-sulfamethoxazole	1 (2)	0	1 (7.7)	0	0	0	2 (2.9)	0
Streptogramins	Quinupristin/Dalfopristin Dalfopristin (QD)	2 (4)	0	1 (7.7)	0	0	0	3 (4.4)	0
Beta-lactam	Penicillin	18 (36)	0	12 (92.3)	0	5 (100)	0	35 (51.5)	0
Ansamycins	Rifampicin	6 (12)	0	0	0	0	0	6 (8.8)	0
Oxazolidinones	Linezolid	0	1 (2)	0	0	0	0	0	1 (1.5)
Lincosamides	Clindamycin	0	0	1 (7.7)	0	0	0	0	0
Fluoroquinolones	Levofloxacin	3 (6)	10 (20)	5 (38.5)	2 (15.4)	0	0	8 (11.8)	12 (17.6)
	Ciprofloxacin	10 (20)	10 (20)	1 (7.7)	6 (46.2)	0	0	11 (16.2)	16 (23.6)
Aminoglicosydes	Gentamycin	31 (62)	1 (2)	7 (53.8)	0	5 (100)	0	43 (63.2)	1 (1.5)
Macrolides	Erythromycin	29 (58)	0	8 (61.5)	0	5 (100)	0	42 (61.8)	0

Overall, 10 strains showed resistance against at least 1 class of antibiotic, and two strains, namely *S. epidermidis* 2.3 and *S. aureus* M14, were resistant to 6 and 5 antibiotics, respectively. Within the *S. aureus* species, 62%, 58%, and 36% of tested strains were resistant to gentamicin, erythromycin, and penicillin, respectively. All tested *S. aureus* strains showed susceptibility towards trimethoprim-sulfamethoxazole, quinupristin-dalfopristin, and clindamycin, and the remaining 98% to linezolid. Moreover, towards levofloxacin and ciprofloxacin, some strains (19.6%) showed an intermediate susceptibility (Table 3). Regarding *S. xylosus*, most strains showed sensitivity to the most analysed antibiotics, except for penicillin, gentamicin, and erythromycin, for which all strains were resistant (Table 3). Compared to the *S. aureus* species, the *S. epidermidis* group showed the highest antimicrobial resistance rate, mainly against penicillin (92.3%), erythromycin (61.5%), gentamicin (53.8%), and a lower percentage even towards ciprofloxacin, trimethoprim-sulfamethoxazole, and quinupristin-dalfopristin molecules (Table 3). Furthermore, the *S. epidermidis* strains, harbouring the *mec* genes, were the most phenotypically resistant. The antimicrobial resistance profile across different clusters of the same species revealed differences. Indeed, only one cluster of *S. aureus* exhibited the same profile for all tested strains, whereas the most numerous clusters showed the most diverse antimicrobial resistance patterns. Similar behaviour was observed within the *S. epidermidis* species. On the contrary, all strains of *S. xylosus* displayed the same antimicrobial resistance profile.

## Discussion

This study aimed to explore and characterize the bacterial microbiota of the healthy eye to assess the biodiversity of culturable bacteria. Results indicated a distinct dominance of the staphylococci group, particularly *S. aureus*, with CoNS following behind. These results highlight that *S. aureus* is the most frequently isolated species on the ocular surfaces of healthy adults [1, 4] and corroborate the low microbial biodiversity present in this area of the human body [7, 23].

Among CoNS, both *S. epidermidis* and *S. xylosus* were the most frequently isolated bacteria, mainly in the conjunctiva, consistent with earlier observations [32]. As a matter of fact, staphylococci are considered key components of human microbiota.

Historically, *S. epidermidis* has been considered less virulent than *S. aureus*. However, accumulating evidence indicates that *S. epidermidis* can act as an opportunistic pathogen, primarily because of its ability to form biofilms and its growing resistance to antimicrobial agents [32]. Our findings support this observation, as all tested strains exhibited resistance to multiple antibiotics, particularly penicillin, erythromycin, and gentamicin. This widespread antimicrobial resistance may contribute to the enhanced pathogenic potential of *S. epidermidis*, as reflected in the growing prevalence of antibiotic-resistant strains in the human ecosystem and clinical settings [21, 22, 40]. However, a large proportion of isolates remained susceptible to trimethoprim/sulfamethoxazole and chloramphenicol, suggesting these antibiotics could be effective options for treating difficult eye infections [3]. Conversely, both *S. aureus* and CoNS showed moderate to high levels of resistance to fluoroquinolones, aminoglycosides, and tetracycline. Notably, multidrug resistance was common, especially among isolates resistant to penicillin and erythromycin. Given that fluoroquinolones, aminoglycosides, and chloramphenicol are widely used to treat ocular infections, the observed resistance profile, together with the increasing prevalence of MRSA strains, substantially narrows the range of effective therapeutic options and poses a significant challenge to the clinical management of staphylococcal ocular infections [2].

PFGE analysis revealed substantial genetic heterogeneity among the isolates, particularly within *S. aureus* and *S. epidermidis* populations. In contrast, the persistence of a dominant *S. xylosus* clone supports its stable association with the indigenous microbiota [41]. Regarding *S. aureus*, results demonstrated a diverse mix of lineages, reflecting high patient-related biodiversity.

The occurrence of identical PFGE types among *S. aureus* and *S. epidermidis* isolates recovered from different individuals may reflect the circulation of successful lineages that are well adapted to the local ecological niche and broadly distributed within the community. Notably, isolates sharing the same PFGE profile displayed different antimicrobial resistance phenotypes, underscoring the considerable genetic plasticity of staphylococci. Such plasticity facilitates adaptation to diverse environmental conditions and promotes the acquisition of traits that may enhance persistence and survival [25, 42].

Finally, the observation that isolates sharing the same PFGE profile displayed different antimicrobial resistance patterns highlights the considerable genetic plasticity of staphylococci. Such plasticity facilitates adaptation to diverse ecological niches through the acquisition, loss, or modulation of genetic determinants associated with antimicrobial resistance and other adaptive traits [25, 42].

## Conclusion

The present study revealed that staphylococci are the predominant cultivable bacterial group within the ocular microbiota in healthy enrolled persons living in Sicily. However, the composition of the microbiota residing on the healthy ocular surface is affected by many factors (such as environment, diet, sex, and age); in the present study, only staphylococci were isolated. While the established role of staphylococci as opportunistic pathogens and the increasing prevalence of antimicrobial resistance warrant continued attention, the low frequency of virulence-associated determinants, including *mec* and *pvl*, indicates that most isolates exhibit limited pathogenic potential. Nevertheless, the composition and functional role of the healthy ocular microbiota remain incompletely characterized [43]. Further investigations into the ecology and dynamics of the ocular microbial community may enhance our understanding of ocular surface homeostasis and provide new insights into the epidemiology and pathogenesis of ocular diseases. Furthermore, considering the substantial genetic diversity of *S. aureus* and *S. epidermidis* and their increasing success in colonizing diverse environmental niches, further research is warranted to determine the safety and clinical relevance of the identified lineages. A deeper understanding of the genetic and phenotypic characteristics of these strains could contribute to the development of targeted and personalized interventions for the maintenance of ocular surface homeostasis and the prevention of ocular disease.

## Supplementary Information

Below is the link to the electronic supplementary material.

Supplementary Material 1 (DOCX 14.9 KB)

## Data Availability

Data supporting the findings of this study are available from the corresponding author upon reasonable request for academic purposes only.
