# Supplementary material for: Phenotypic characterization, molecular typing, and clonal relatedness within Staphylococcus isolates in healthy ocular conditions
Source: Braz J Microbiol. 2026 Jun 24;57(1):182. doi: 10.1007/s42770-026-01999-5 (PMC13294426; doi:10.1007/s42770-026-01999-5)
Supplement: Supplementary file 1 — Supplementary Material 1 (DOCX 14.9 KB) [file 42770_2026_1999_MOESM1_ESM.docx]

| Primer | Primer # (EURL-AR) | Sequence | Amplicon size (pb) |
| --- | --- | --- | --- |
| *spa*-1113F | 2819 | 5’–TAAAGACGATCCTTCGGTGAGC–3’ | 180-600 |
| *spa*-1514R | 2820 | 5’–CAGCAGTAGTGCCGTTTGCTT–3’ |  |
| *mecA* P4 | 2821 | 5’ –TCCAGATTACAACTTCACCAGG–3’ | 162 |
| *mecA* P7 | 2822 | 5’– CCACTTCATATCTTGTAACG–3’ |  |
| *pvl*-F | 2823 | 5’– GCTGGACAAAACTTCTTGGAATAT–3’ | 83 |
| *pvl*-R | 2824 | 5’–GATAGGACACCAATAAATTCTGGATTG–3’ |  |
| *mecA*_LGA251_ MultiFP | 2825 | 5’– GAAAAAAAGGCTTAGAACGCCTC–3’ | 138 |
| *mecA*_LGA251_ MultiRP | 2826 | 5’–GAAGATCTTTTCCGTTTTCAGC–3’ |  |

**Table 1S.** Primer sequences and amplicon sizes
